# Supplementary figures and images for: Colon Cancer Microbiome Landscaping: Differences in Right- and Left-Sided Colon Cancer and a Tumor Microbiome-Ileal Microbiome Association
Source: Int J Mol Sci. 2023 Feb 7;24(4):3265. doi: 10.3390/ijms24043265 (PMC9963782; doi:10.3390/ijms24043265)

# Random Forest Classification

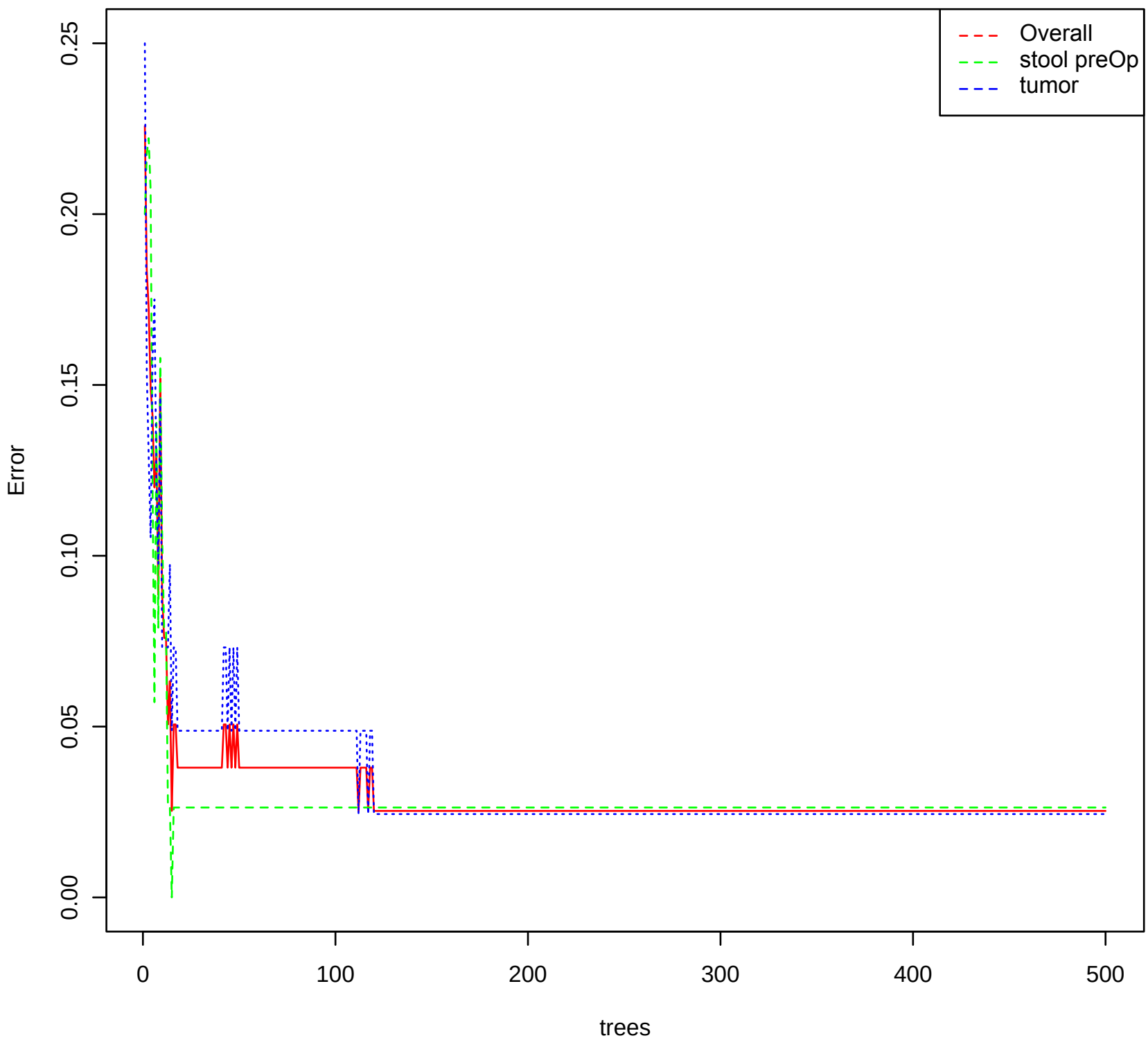

Supplement: Supplementary file 1 [file ijms-24-03265-s001.zip › Supplementary Figure S1-Random forest classification machine learning algorithm to discriminate between stool and tumor samples.pdf]

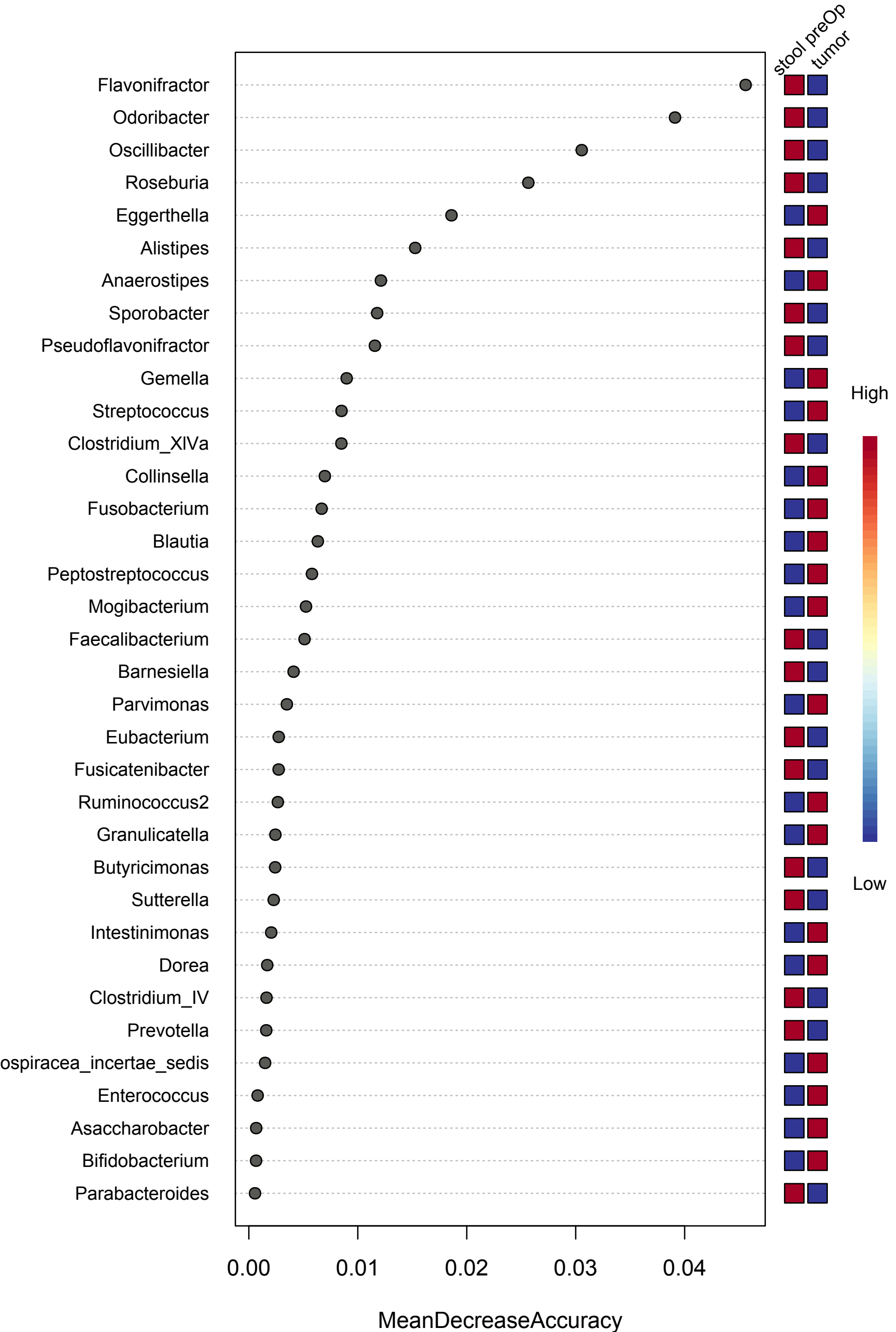

Supplement: Supplementary file 1 [file ijms-24-03265-s001.zip › Supplementary Figure S2-Top genera to discriminate between stool and tumor samples.pdf]

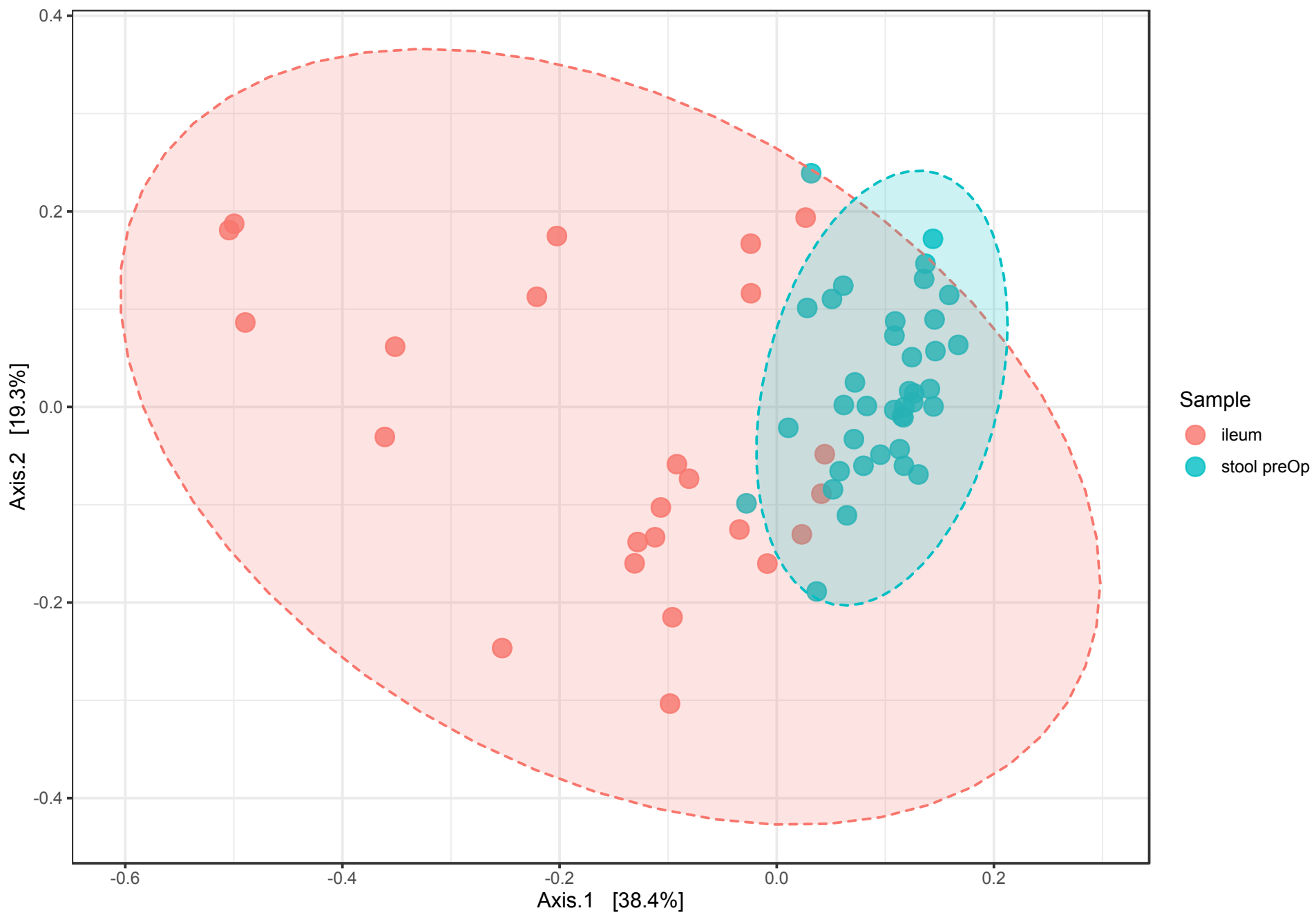

Supplement: Supplementary file 1 [file ijms-24-03265-s001.zip › Supplementary Figure S3-Beta diversity (PCoA Jensen¿CShannon) analysis between ileal and stool samples.pdf]

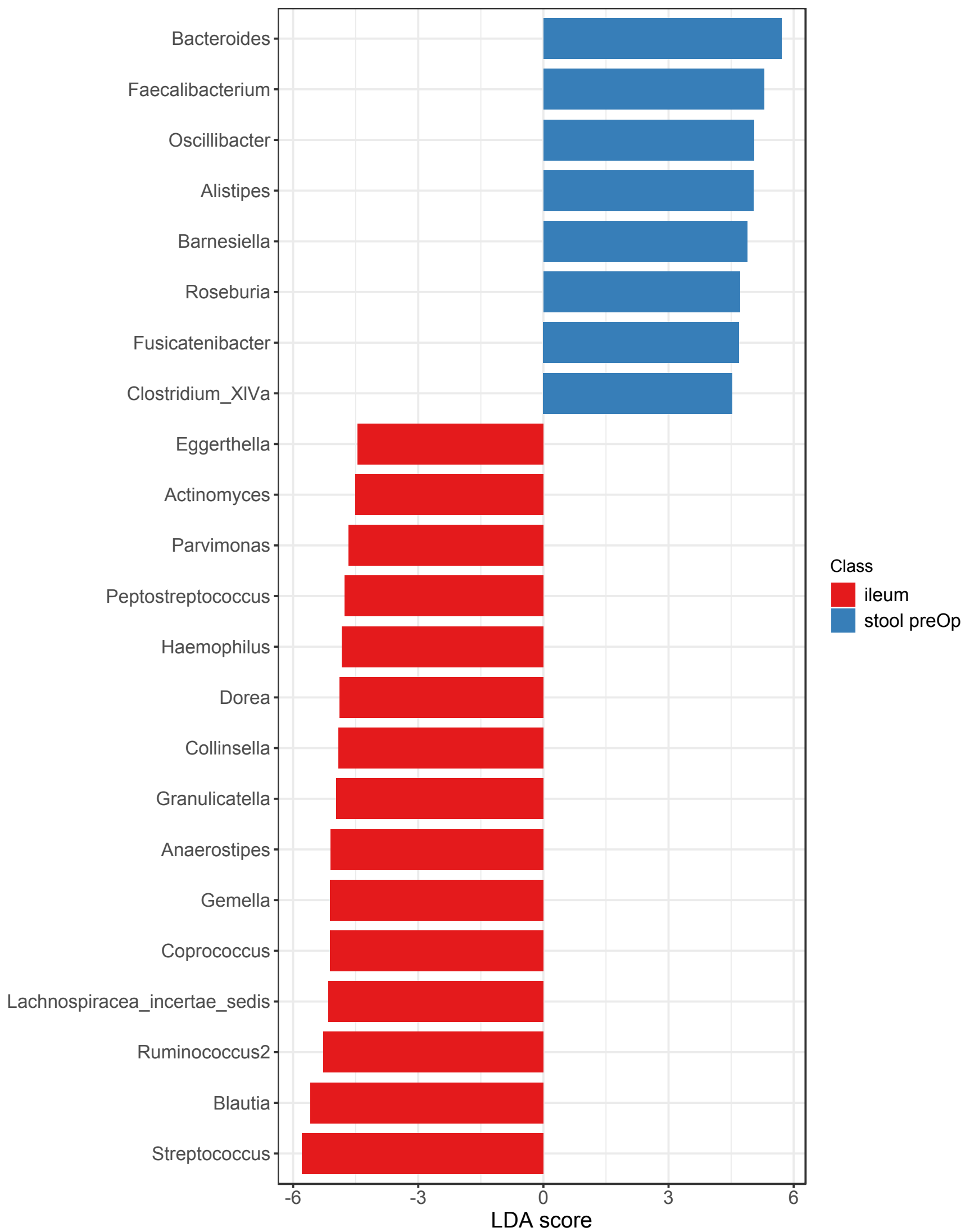

Supplement: Supplementary file 1 [file ijms-24-03265-s001.zip › Supplementary Figure S4-LEfSe analysis of ileal and stool samples.pdf]
